# Supplementary material for: Identification of photoperiod-induced specific miRNAs in the adrenal glands of Sunite sheep (Ovis aries)
Source: Front Vet Sci. 2022 Jul 22;9:888207. doi: 10.3389/fvets.2022.888207 (PMC9354845; doi:10.3389/fvets.2022.888207)
Supplement: Supplementary file 1 [file Data_Sheet_1.ZIP › Supplementary file/miRDeep2 file.zip!/miRDeep2 file/Know_Structure/LP42A2/oar-mir-411b.pdf]

miRBase precursor : oar-mir-411b
Total read count : 2529
oar-miR-411b-5p read count : 2498
oar-miR-411b-3p read count : 27
remaining reads : 4

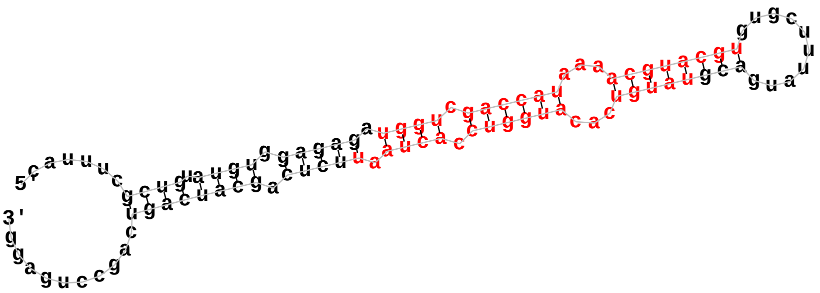

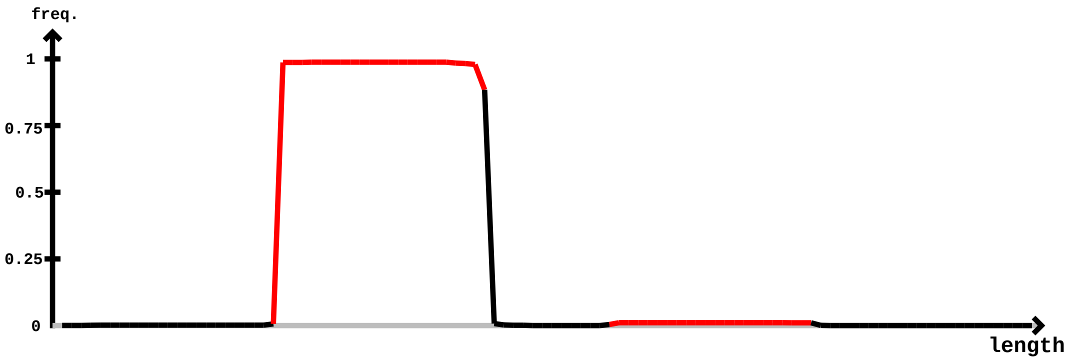

oar-miR-411b-3p

oar-miR-411b-5p

5'- cauuucgcuguauguggagagauggucgaccauaaaaacguacgugugcuuuaugacguaugucacaugguccacuaauucucagcaucagucagccugagg -3'	exp		
......((((.((((.(((((.((((.((((((...((((((((..........))))))))...)))))).)))).))))).)))))))))..........	reads	mm	sample
cauuucgcuguauguggagaga..	1	0	seq
..uucgcuguauguggagag..	1	0	seq
..uucgcuguauguggagaga...	1	0	seq
...ucgcuguauguggagaga...	1	0	seq
.....................auggucgaccauaaaaacguac...	1	0	seq
.....................auggucgaccauaaaaacguacg..	12	0	seq
......................uggucgaccauaaaaacgu...	9	0	seq
......................uggucgaccauaaaaacgua..	4	0	seq
......................uggucgaccauaaaaacguac...	8	0	seq
......................uggucgaccauaaaaacguacg..	228	0	seq
......................uggucgaccauaaaaacguacgu...	2217	0	seq
......................uggucgaccauaaaaacguacgug..	13	0	seq
......................uggucgaccauaaaaacguacgugu...	3	0	seq
.........................ucgaccauaaaaacguacgugugc...	3	0	seq
....guaugucacaugguccacuaau.......................	10	0	seq
....uaugucacaugguccacu..........................	1	0	seq
....uaugucacaugguccacuaau.......................	14	0	seq
....uaugucacaugguccacuaauu......................	2	0	seq
